# Powdered Ceramsite and Powdered Limestone Use in Aerobic Granular Sludge Technology

**DOI:** 10.3390/ma13173894

**Published:** 2020-09-03

**Authors:** Joanna Czarnota, Janusz A. Tomaszek, Adam Masłoń, Adam Piech, Grzegorz Łagód

**Affiliations:** 1 Department of Environmental Engineering and Chemistry, Rzeszow University of Technology, 6 Powstańców Warszawy Av, 35-959 Rzeszów, Poland; tomaszek@prz.rzeszow.pl (J.A.T.); amaslon@prz.edu.pl (A.M.); 2 Department of Water Purification and Protection, Rzeszow University of Technology, 6 Powstańców Warszawy Av, 35-959 Rzeszów, Poland; apiech@prz.edu.pl; 3 Faculty of Environmental Engineering, Lublin University of Technology, Nadbystrzycka 40B, 20-618 Lublin, Poland

**Keywords:** powdered ceramsite, powdered limestone, AGS technology, organic loading rate, wastewater treatment

## Abstract

The effects of two powdered mineral materials (powdered ceramsite and powdered limestone) on aerobic granulation of sludge were evaluated. The experiment was conducted on a laboratory scale bioreactors treating wastewater for 89 days. Three granular sequencing batch reactors (GSBRs) were operated at the lowest optimal organic loading rate (OLR) of 2.55 g COD/(L∙d). In the control reactor (R1), the mean diameter (d) of the biomass ranged from 124.0 to 210.0 µm, and complete granulation was not achieved. However, complete granulation did occur in reactors to which either ceramsite (251.9 µm < d < 783.1 µm) or limestone (246.0 µm < d < 518.9 µm) was added. Both powdered materials served as a ballast for the sludge flocs making up the seed sludge. Ceramsite particles also acted as microcarriers of granule-forming biomass. The granules in the reactors with added powdered materials had nonfibrous and smoother surfaces. The reactor with ceramsite exhibited the highest average efficiencies for COD, total nitrogen, and total phosphorus removal (85.4 ± 5.4%, 56.6 ± 10.2%, and 56.8 ± 9.9%, respectively). By contrast, the average nitrification efficiency was 95.1 ± 12.8%.

## 1. Introduction

The use of aerobic granular sludge (AGS) is a promising and relatively new technology for the treatment of different types of wastewater; it allows for the simultaneous removal of carbon, nitrogen, phosphorus, and other pollutants in a single reactor [1,2,3]. Importantly, the operating conditions supporting aerobic granulation are strongly determined by various parameters, including the reactor configuration, seed sludge, settling time, organic loading rate (OLR), volume exchange ratio, hydrodynamic shear force, feast–famine regime, cycle time, and other environmental conditions (notably, pH and temperature) [4,5,6]. Most reports on successful granulation have shown that a high OLR is critical to the development and maturation of aerobic granules [7,8,9]. Higher OLR facilitates granule formation [9,10,11,12]. According to a literature review, the optimal OLR of a granular sequencing batch reactor (GSBR) is 2.50 g COD/(L∙d) [13] or should be in the range 2.50–7.50 g COD/(L∙d) [14,15]. In treatment of wastewater with a lower chemical oxygen demand (COD) concentration, a higher organic load can be obtained by shortening the cycle time [10,16]. At a low OLR, sludge granulation also requires a relatively long startup period (more than three months) [10]. Despite considerable research, new methods and means of improving AGS technology are still being sought. The objective is to improve biogranulation and stability of aerobic granules, especially through improved AGS formation in low-strength wastewater [6,14].

To date, research on improving AGS technology has involved chemical coagulants [17], dosage of granule fragments [18,19], and powdered materials, both mineral (Table 1) [4,11,20,21,22] and organic [10,23,24,25,26,27,28]. It should be emphasized that in addition to the use of metal ions and flocculants, organic materials are most often described in the latest review articles [29,30], the subject of which concerns strategies implemented to improve the biogranulation process.

According to the literature, powdered materials added to a GSBR should have a sufficiently large specific surface area and be sufficiently heavy and spherical. Moreover, they should settle out rapidly enough and also be stably suspended with biomass in the reactor [15,31]. In AGS technology, these materials (both mineral and organic) are microcarriers of biomass that forms aggregates [15,31]. Additionally, the use of mineral and organic powdered materials can shorten the time required for activated sludge to granulate because they act as nuclei that induce the aggregation of microorganisms [25]. Furthermore, divalent cations (i.e., Ca^2+^, Mg^2+^) in the mineral materials may participate in the formation of aggregate matrices [32,33].

Application of powdered materials in AGS technology causes more rapid biomass sedimentation, reduces the time required for biogranulation, and decreases the amount of total suspended solids at the outlet. Furthermore, it significantly impacts lower values for the sludge volume index (SVI), granules density, and stability of granules during long-term reactor operation [21,24,25,34].

The main aim of this work was to evaluate the reasonability of powdered ceramsite and powdered limestone use in the aspect of improving the aerobic granular sludge technology. Compared to state-of-the-art materials, the novelty of the conducted research should be emphasized, because it allows us to compare the impacts of two different and hitherto unused powdered mineral materials on AGS technology. Using statistical analysis, a comparison of biomass parameters and efficiency of wastewater treatment in two reactors with addition of different powdered mineral materials in relation to the control reactor was made. In addition, the relationships between selected biomass parameters in individual reactors were determined, which clearly confirmed that use of these materials is reasonable. Note that the experiments were conducted in GSBRs with the lowest optimal OLR. Our own preliminary research on the application of powdered ceramsite in AGS technology is described in the article Czarnota et al. [15].

## 2. Materials and Methods

### 2.1. Powdered Materials

The study used two powdered mineral materials, ceramsite (PK) and limestone (PL). Powdered ceramsite is a waste fraction from the production of ceramic aggregates, and its natural grain size ranges from 0.39 to 200 µm [11,15,33]. A powdered fraction of limestone was obtained by grinding a coarse material mechanically and then sieving it through a 0.2 mm sieve. The selected powdered materials were characterized in terms of various physical and chemical properties (Table 2). The present study uses materials in which smaller particles are dominant. The specific surface areas of these materials were smaller than the surface areas of materials (especially organic materials) used in the previous AGS research. The particles were assumed to function mainly as a ballast for sludge flocs, with only a limited role as a biomass microcarrier. In addition, the analysis followed up on previous reports [35,36,37] of a positive impact of cations such as Ca^2+^, Mg^2+^, and Fe^3+^ on the biogranulation process.

### 2.2. Reactor Set-Up and Operating Strategy

Three lab-scale GSBRs with 3.0 L working volume were operated in parallel for 89 days. The parameters of experiment (geometrical configuration of reactors, flow rate, volumetric exchange ratio, hydraulic retention time, air supply, and cycle of operation) were adopted according to studies of Czarnota et al. [15]. The technical parameters of the reactors are presented in Table 3.

**Figure 1:**
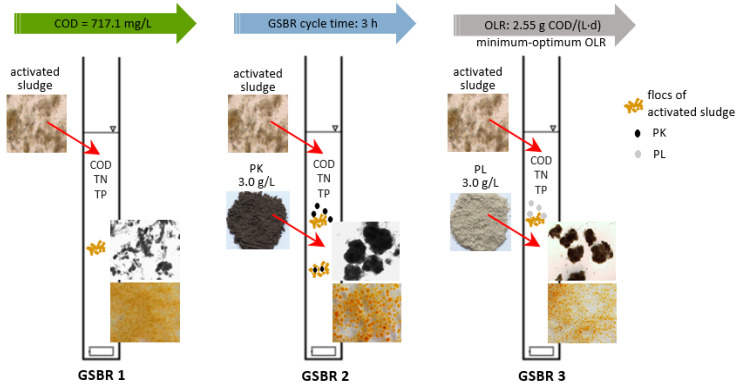
Graphic representation of the way the research was conducted.

The first GSBR (R1—without powdered material) was a control reactor, whereas 3.0 g/L of PK or PL were added to reactors R2 and R3, respectively (Figure 1). The construction of a single reactor used in the research has been presented in the article by Czarnota et al. [33].

The airflow velocity in the reaction phase (aeration) was 0.8 cm/s, and rotameters were used to adjust this parameter. The reactors were operated automatically using time controllers. The temperature was about 20 °C during the entire experiment.

In order to start lab-scale GSBRs, activated sludge from municipal wastewater treatment plant in Rzeszów (Poland) was drawn. The inoculum had following parameters: initial mixed liquor suspended solids (MLSS) about 6.40 g MLSS/L and sludge volume index (SVI_30_) about 135.2 mL/g. The volume of inoculum was 1.8 L/volume of reactor, and the same volume was used in our own further research [11].

The study was performed using synthetic wastewater, which was prepared according to the chemical composition given by Thanh et al. [21]. The composition was as follows: glucose (664.0 mg/L), NaHCO_3_ (450.0 mg/L), NH_4_Cl (150.0 mg/L), KH_2_PO_4_ (43.0 mg/L), CaCl_2_∙2H_2_O (30.0 mg/L), MgSO_4_∙7H_2_O (12.0 mg/L), FeCl_3_ (3.6 mg/L), and 1.0 mL/L of trace elements. The major sources of phosphorus, nitrogen, and organic compounds were potassium dihydrogen phosphate, ammonium chloride, and glucose, respectively. The experiment was conducted using various concentrations of pollutants in the influent (Table 4).

The COD value in the synthetic wastewater was 717.1 ± 62.6 mg O_2_/L, the same values figure in article Czarnota et al. [15]. Calculated OLR was approximately 2.55 g COD/(L·d); this is the minimum value of the organic compound load that can be considered optimal for AGS technology.

### 2.3. Analytical Method

The parameters of raw and treated wastewater, such as the COD, total nitrogen (TN), total Kjeldahl nitrogen (TKN), ammonium-nitrogen (NH_4_^+^–N), nitrite-nitrogen (NO_2_^−^–N), nitrate-nitrogen (NO_3_^−^–N), total phosphorus (TP), and phosphate-phosphorus (PO_4_^3^^−^–P), were determined using photometric measurement methods according to Polish Standards (PN). These parameters were analyzed using commercial Hach Lange^®^ test kits (for COD; Hach Berlin, Berlin, Germany), Spectroquant^®^ Reagent Test Kits (for TN, NH_4_^+^–N, NO_2_^−^–N, NO_3_^−^–N, TP and PO_4_^3^^−^–P; Merck KGaA, Darmstadt, Germany), and spectrophotometers (Hach DR5000 for COD and Aquamate Thermo Spectronic for other parameters, Hach Berlin, Berlin, Germany and Thermo Fisher Scientific, Waltham, MA, USA). The dissolved oxygen (DO) and pH were also monitored using a DO meter (1-channel multimeter Hach HQ30d with sonde, Hach Berlin, Berlin, Germany) and pH meter (1-channel multimeter Hach HQ30d with pH electrode, Hach Berlin, Berlin, Germany). The biomass parameters, such as sludge volume index (SVI_5_ and SVI_30_), sludge settling velocity (V), MLSS, and mixed liquor volatile suspended solids (MLVSS) were determined according to standard methods [38]. The specific oxygen uptake rate (SOUR) for biomass was determined using a methodology described by Kristensen et al. [39] and Zielińska et al. [40]. Optical microscopy (Olympus BX51, Olympus America, NY, USA) was used to observe the sludge floc and aerobic granule morphology. A method proposed by Arrojo [41], which uses the specialized CellQ software (standard version, Olympus America, NY, USA), was used to determine the aerobic granule size. The percentage share of the individual fractions (the smallest fraction is d < 200 µm) was determined by relating the number of granules in a given observation range to the total number of observations (minimum 200 granules from one reactor). By calculating the average value for the granules diameters on a given day, the average granule size (d) was determined. Granular structure was identified by scanning electron microscopy (SEM) (MIRA Tescan, Tescan Orsay Holding, Brno, Czech Republic). Samples of biomass were lyophilized before the SEM analysis. In addition, computed tomography (Computer Tomograph Phoenix v|tome|x m, Baker Hughes a GE Company, Houston, TX, USA) was used to assess differences on the granule surfaces. Measurements were made using frozen samples of biomass that were insulated using a layer of styrofoam.

### 2.4. Statistical Analysis

STATISTICA 10 PL software (version 10 PL, TIBCO Software Incorporation, Palo Alto, CA, USA) was used for statistical analysis. Using Pearson’s linear correlation, the relationships between pairs of variables were evaluated, and the statistical significance was determined. A significance level *α* of 0.05 was used. The Kruskal–Wallis ANOVA test was used to evaluate the differences between the averages of several groups. The probability of error related to the assumption of the hypothesis that there are differences between the averages was assumed at 5% (*p* < 0.05).

## 3. Results and Discussion

### 3.1. Biomass Concentration in the Reactors

**Figure 2:**
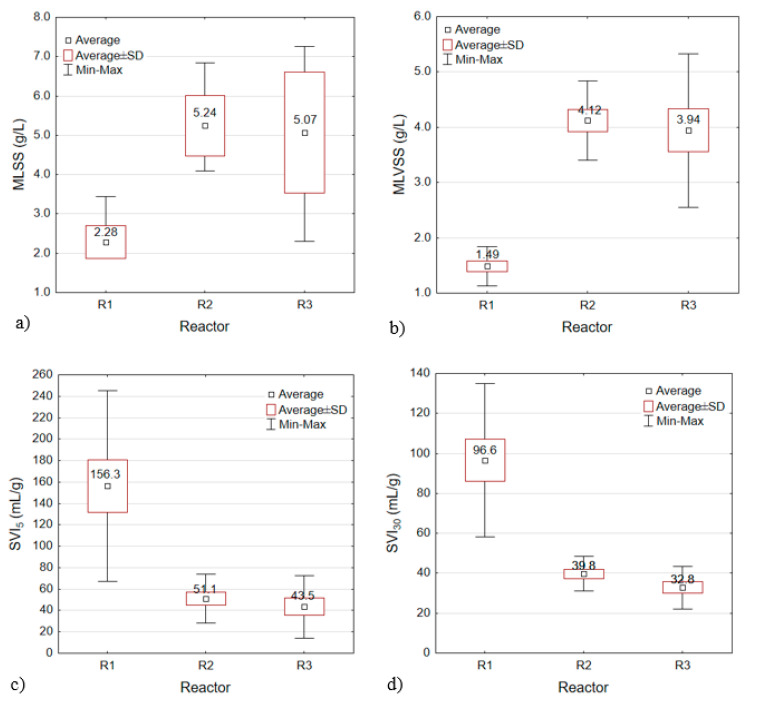
Average MLSS (**a**), MLVSS (**b**), SVI_5_ (**c**), and SVI_30_ (**d**) values of reactors.

The MLSS after inoculation was 3.90 g/L in each GSBR. Application of powdered materials (ceramsite in R2 and limestone in R3) increased this value to 7.38 g MLSS/L in R2 and 5.39 g MLSS/L in R3. The MLSS values measured after the five-day adaptation period were 46%, 43%, and 28% lower than the initial values in R1, R2, and R3, respectively. The MLSS values of R2 and R3 then increased, reaching average values of 5.24 ± 0.78 and 5.07 ± 1.54 g/L, respectively (Figure 2a). The MLVSS concentrations in R1, R2, and R3 decreased by 44.5%, 22%, and 19%, respectively, during the adaptation period. Further, the average MLVSS values after the adaptation period were 1.49 ± 0.36, 4.12 ± 0.71, and 3.94 ± 1.39 g/L, respectively (Figure 2b). The short settling time in the GSBR cycle (4 min) favored the elution of biomass from the reactors, but the results still showed better retention in the reactors with powdered mineral materials than in the control reactor. Control of the biomass concentration in R1 necessitated control of the hydraulic retention time. The MLSS and MLVSS values were much higher in reactors R2 and R3 than in R1, and analysis showed that the differences in both the MLSS and MLVSS values of the reactors reached statistical significance (*p < α*, *p* = 0.0000 for MLSS, *p* = 0.0000 for MLVSS). Of the two reactors operating with powdered materials, R2 exhibited the greatest stability during the tests. The increased biomass concentrations in reactors R2 and R3 were found to reflect increases in both the size and number of granules.

Following inoculation of the reactors, the MLVSS/MLSS ratio was approximately 0.74. The addition of mineral material caused that ratio to decrease to 0.53 (R2) and 0.68 (R3). After the twelfth day, when biogranulation was observed, the MLVSS/MLSS ratio was 0.76–0.84 in R2 and 0.67–0.84 in R3, indicating increases in the bioactivity of the sludge. In the control reactor, this parameter did not increase until after day 19 and reached a maximum value of 0.77.

### 3.2. Settling Properties of the Biomass

The initial SVI_5_ and SVI_30_ values of the control reactor were 240.3 and 152.2 mL/g, respectively. By contrast, these values were significantly lower when the powdered materials were applied. In reactors R2 and R3, the initial SVI_5_ values were 116.5 and 166.0 mL/g, and the initial SVI_30_ values were 52.2 and 82.6 mL/g, respectively. By the end of the study, the SVI_5_ values in R1, R2, and R3 were 108.8, 30.7, and 26.1 mL/g, and the SVI_30_ values were 92.1, 30.7, and 21.7 mL/g, respectively. The average SVI_5_ and SVI_30_ values are presented in Figure 2c,d, respectively. The reactor with powdered ceramsite (R2) had the most stable sludge volume, as it showed the lowest coefficient of variation for this parameter. The results of this experiment are consistent with, for example, the results reported by Li et al. [24], who found that the SVI_30_ values in a reactor with initial granular activated carbon (GAC) addition were lower throughout the process than those in a reactor without GAC. Other authors also observed that powdered materials decreased the SVI and improved the stability of granules over a prolonged period of system operation (Table 5). By contrast, Tao et al. [26] reported that the SVI_30_ values of two reactors (without or with GAC) did not obviously differ in or after the granulation phase. An extensive discussion of the results of own research with the literature data in this area is detailed in the publication by Czarnota et al. [33].

**Figure 3:**
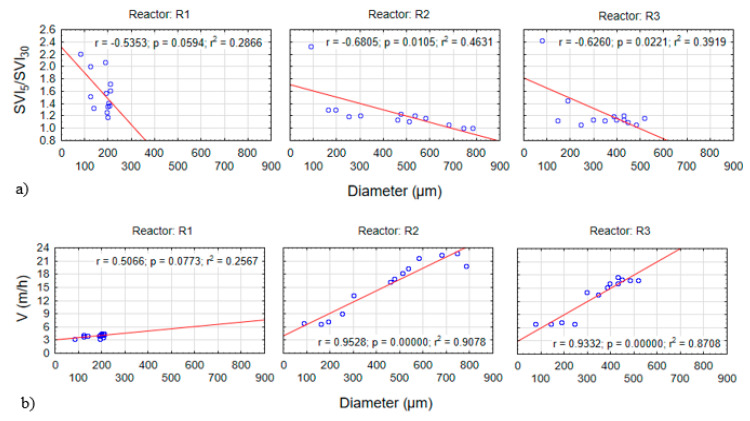
Scatter graphs showing the relationships between the granule diameter and SVI_5_/SVI_30_ (**a**) and settling velocity (**b**).

Starting on the nineteenth day of the experiment, the SVI_5_/SVI_30_ ratio was observed to be significantly lower in the reactors with powdered materials. The SVI_5_/SVI_30_ values of R2 and R3 indicate that the addition of PK and PL could clearly improve both the sludge settling capacity and its compressibility. Marked correlations were noted between a lower SVI_5_/SVI_30_ value and increased floc size (early in the experiment) and the growth of granules (later in the experiment). In the reactors with PK and PL, data dependency was found to be statistically significant (*p < α*, *p* = 0.0105 for R2, and *p* = 0.0221 for R3) (Figure 3a).

The application of mineral materials increased the settling velocity. First, in the early stages of the experiment, the materials acted as ballast for sludge flocs, increasing their weight. This affected the sedimentation properties of the biomass and also reduced the rate of biomass elution from reactors R2 and R3, ensuring that biogranulation occurred. Higher settling velocities were observed in reactors R2 and R3 (Figure 3b), and the values resembled those given in the literature. Li et al. [42] showed that, in a reactor to which GAC (0.5 g/L) had been added, the settling velocity of the biomass was 18.5 m/h. However, in a reactor to which powdered activated carbon (PAC) (0.5 g/L) was added, this value was 13.7 m/h. By contrast, Wei et al. [23] reported higher settling velocities of up to 25 m/h, as well as a relationship between the increased velocity and increased density and diameter of the granules. These values are much higher than the settling velocity of ordinary activated sludge, which is 1–2 m/h [21]. The relationship between the settling velocity and granule diameter was statistically significant in the reactors with powdered ceramsite and limestone (*p* < *α*, *p* = 0.0000 for R2, and *p* = 0.0000 for R3) (Figure 3b).

### 3.3. Formation of Aerobic Granules

**Figure 4:**
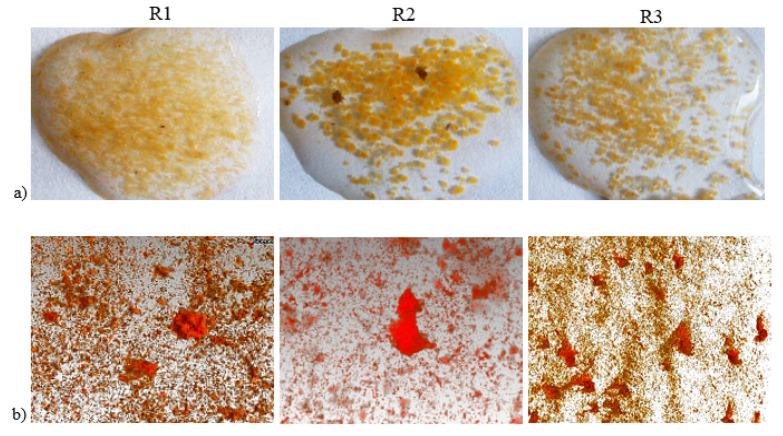
Characteristic biomass: photographs (**a**) and X-ray tomography images (**b**) of granules after 89 days of granulation.

Fourteen days after the acclimation period ended, the cumulative volume percentages of granules with diameters exceeding 200 µm were 9.38%, 38%, and 31% in reactors R1, R2, and R3, respectively. This result indicated that sludge flocs were still the dominant form of biomass in both reactors supplemented with powdered mineral. The mean aggregate size measured on day 26 had increased from 76.4 to 246.0 µm (R3) and from 89.3 to 251.9 µm (R2). After 54 days, complete granulation was observed (according to the definition [19], when particles smaller than 200 µm constitute less than 10% of biomass, full granulation is said), and the mean aggregate size was 511.3 µm in R2 and 396.0 µm in R3. No apparent increase in granule size was observed in R1. After 89 days of GSBR operation, the mean sizes of the aggregates were 200.2, 783.1, and 430.0 µm in R1, R2, and R3, respectively (Figure 4a). The biogranulation process itself with the addition of these two materials, among others, is discussed in more detail in the article by Czarnota et al. [33], which presents the impact of different powdered mineral materials on selected properties of aerobic granular sludge. Table 5 lists the average sizes of granules reported in previous studies of reactors in which powdered materials were applied. An extensive discussion of the results of own research with the literature data in this area is detailed in the publication by Czarnota et al. [33].

The more stable growth of granules in reactor R2, as well as the presence of larger aggregates, resulted from the application of ceramsite. Under a microscope, ceramsite particles were clearly visible within structures formed by sludge flocs and granules (regular suspending together with the biomass). To a lesser extent, the same was also observed for the powdered limestone. In addition, it was possible to observe the formation of granules containing microparticles of ceramsite. More information on these issues may be found in the previous publication [33] of the authors. Furthermore, after 68 days, the average size of granules in reactor R3 was decreasing, which is consistent with results reported by Wei et al. [23]. In a reactor with added PAC, on the fiftieth day of testing, the average granule size was 364.0 µm, and the percentage of aggregates above 200.0 µm in diameter was 67.1%. In the following days, the authors observed an increase in granule diameter compared with that on the fiftieth day. However, the average granule size decreased over time (from 596.0 µm on day 90 to 485.0 µm on day 113). The percentage of aggregates with sizes above 200.0 µm increased to a maximum of 79.6%. In the control reactor, 50 days into the research, the granules began to disintegrate. The authors summarized the research by saying that the application of PAC afforded a stable biogranulation process, and the obtained granules showed greater stability and more favorable sedimentation properties.

**Figure 5:**
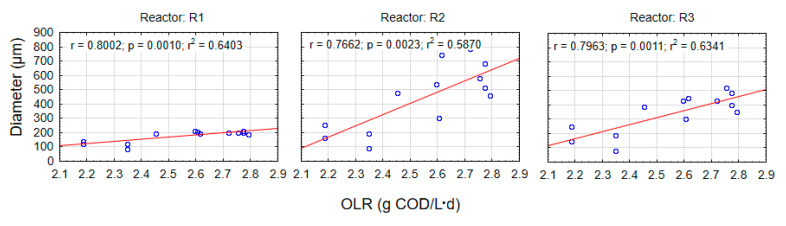
Scatter graph showing the relationship between granule diameter and OLR.

Statistical analysis revealed a highly significant relationship between a slight increase in OLR and granule growth (*p* < *α*, *p* = 0.0010 for R1, *p* = 0.0023 for R2, and *p* = 0.0011 for R3) (Figure 5). Even though the use of the lowest optimal OLR, with an average value of 2.55 g COD/(L·d), affected the volume of the reactor, biogranulation occurred slowly in the control system. The powdered materials can thus be said to have played an important role in the biogranulation process.

### 3.4. Structural Characteristics and Bioactivity of Granular Sludge

The computed tomography results revealed significant differences between the surfaces of granules from different reactors. In the control reactor, the biomass had a fluffy surface with filamentous microorganisms. These bacteria dominated the granules and contributed to loosening of their structure [43]. By contrast, the granules from the reactors with added powdered materials exhibited nonfibrous and smoother surfaces. The surfaces of the granules from reactors R2 and R3 had visible corrugations, most probably because of the greater porosity of these aggregates (Figure 4b).

The application of powdered ceramsite and limestone prevented biomass from being washed out of the reactors and thus limited the development of filamentous bacteria [33]. The SEM analysis indicates that these bacteria formed a framework around the granules. However, in reactor R1, in which the number of filamentous microorganisms was greatest, they not only formed a framework around the granules, but also filled in the spaces in that framework, which affected the qualitative composition of the biocoenosis in this reactor. By contrast, open spaces were present in the framework of granules in the reactors with powdered materials. Their presence provided for the existence of other microorganisms and filled voids inside the granules.

Ca-rich granules have higher mechanical strength but lower bioactivity, so the calcium ion content of aerobic granules is one of the factors determining their bioactivity. The granulation time and stimulation of cell-to-cell aggregation can be reduced by calcium-induced dehydration of bacterial cell surfaces [43,44,45]. In the present study, the concentration of calcium ions in the raw wastewater was 0.27 mmol Ca/L. Additionally, in the first cycle of reactor operation (under assumed hydraulic conditions), 0.127 mmol Ca/L from the powdered ceramsite or 0.674 mmol Ca/L from the powdered limestone was introduced. The total amount of calcium cations was less than 1.0 mmol Ca/L throughout the experiment. Ren et al. [35] showed that when granules grew on wastewater with a calcium concentration of 1.0 mmol Ca/L, the SOUR was 9.55 ± 0.11 mg O_2_/(g VSS∙h), and when the Ca^2+^ concentration was 0.5 mmol Ca/L, the SOUR was 22.26 ± 0.25 mg O_2_/(g VSS∙h). The rate of oxygen uptake by biomass was highest in R1 and ranged from 20.42 to 28.57 mg O_2_/(g VSS∙h), but on the eighty-second day of the study, the rate decreased significantly to 7.85 mg O_2_/(g VSS∙h). These results indicate insufficient biomass content in the reactor and may also suggest accumulation of compounds (e.g., nitrates) with toxic or inhibitory effects on certain groups of microorganisms in the final stage of the experiment. The SOUR was 9.16–14.96 mg O_2_/(g VSS∙h) in R2 and 6.81–12.65 mg O_2_/(g VSS∙h) in R3. The lower SOUR values in these reactors result mainly from higher biomass concentrations.

### 3.5. Carbon, Nitrogen, and Phosphorus Removal

**Figure 6:**
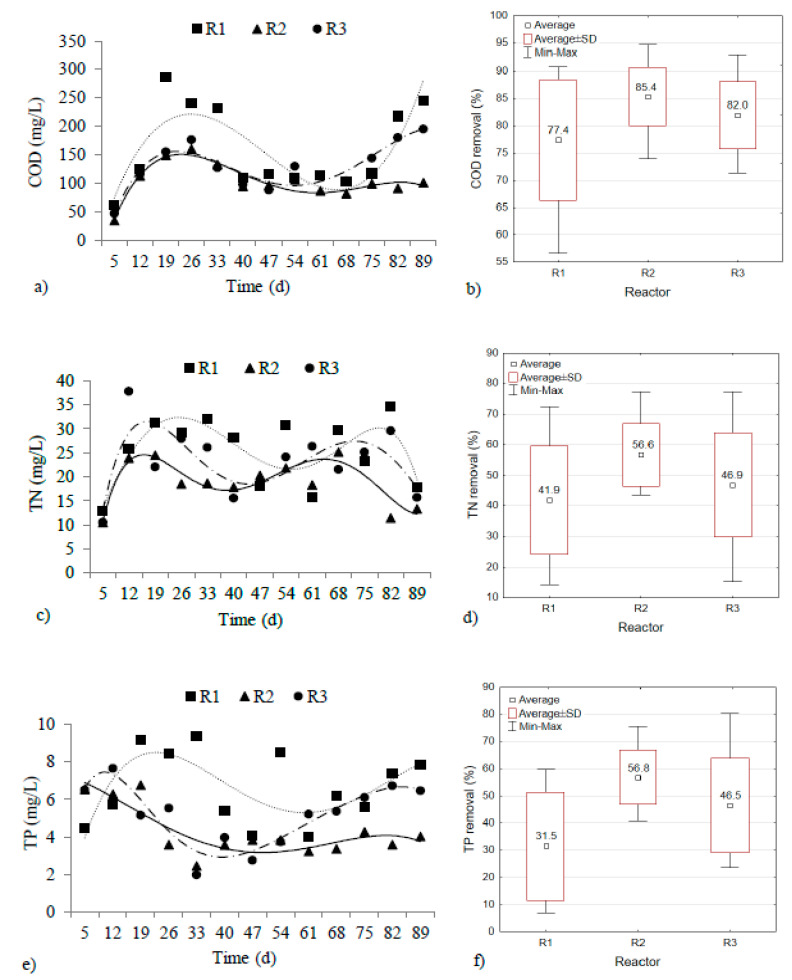
Reactor performance: changes in COD (**a**), TN (**c**), TP (**e**) in effluent and average values of COD (**b**), TN (**d**), and TP (**f**) removal.

The influent COD concentration of the three reactors was 717.1 ± 62.6 mg O_2_/L, and the OLR was approximately 2.55 g COD/(L·d). The COD values in the effluent of R1, R2, and R3 ranged from 60.8 to 286.4 mg O_2_/L, 34.4 to 160.0 mg O_2_/L, and 46.4 to 195.2 mg O_2_/L, respectively (Figure 6a). It was possible to remove 77.4 ± 11.1% of the organic compounds in R1, 85.4 ± 5.4% in R2, and 82.0 ± 6.1% in R3 (Figure 6b). The reactors with powdered materials exhibited better COD removal. The food-to-microorganism (F/M) ratio was 0.757–1.137 g COD/(g VSS∙d) in the control reactor, 0.382–0.676 g COD/(g VSS∙d) in R2, and 0.302–1.183 g COD/(g VSS∙d) in R3. Wu et al. [46] studied the optimal F/M ratio for granule stabilization. They found that F/M ratios between 0.4 and 0.5 g COD/(g VSS·d) were associated with greater microbial diversity and higher pollutant removal efficiency.

Kruskal–Wallis testing of many independent samples did not reveal statistically significant differences in COD removal between the reactors (*p* > *α*, *p* = 0.0534). However, comparison between the value of the control reactor and that of each reactor with a powdered material, and the use of the Mann–Whitney U test for two independent samples, showed statistically significant differences in COD removal between R1 and R2 (*p* < *α*, *p* = 0.0240). The values obtained in R2 were close to those obtained by Minh [20], who used basalt to support a reactor operating at an OLR of 3.0 g COD/(L·d) (78.0–97.0%). The values were also higher than those reported by Wei et al. [23], who applied PAC to a reactor operating at an OLR of 2.10 g COD/(L·d) (approximately 65.0%).

Organic compounds were removed during the feeding and reaction phases of the wastewater treatment cycle. In the reactors with powdered materials, the dynamics of COD removal were higher than those in the control reactor. In the settling, decantation, and idle phases, a slight decrease in the efficiency of organic compound removal was observed, most probably as a result of the release of EPS to the wastewater. Kończak and Miksch [47] studied the dynamics of organic compound removal, as well as changes in the protein and polysaccharide contents during the cycle. They found that the COD concentrations decreased in the aeration phase, during which the extracellular protein content increased and the polysaccharide content remained constant. However, under a prolonged substrate shortage, previously consumed microorganisms accumulated cellular proteins, some of which were released into the supernatant, causing secondary contamination of the treated wastewater with organic substances.

The influent TN concentration of the three GSBRs was 44.0 ± 3.0 mg N/L, and the TN loading rate of the reactors was 0.157 ± 0.011 g N/(L·d). The TN values in the effluent of R1, R2, and R3 were 12.9–34.6 mg N/L, 10.6–25.2 mg N/L, and 10.5–37.8 mg N/L, respectively (Figure 6c). The average efficiencies of TN removal were highest in reactor R2 (56.6 ± 10.2%) compared with R1 and R3 (41.9 ± 17.7% and 46.9 ± 17.1%, respectively) (Figure 6d). Kruskal–Wallis testing of many independent samples did not reveal statistically significant differences in TN removal between the reactors (*p* > *α*, *p* = 0.0943). However, a comparison of the control reactor and each reactor with a powdered material, and application of the Mann–Whitney U test to two independent samples, showed statistically significant differences in TN removal between R1 and R2 (*p* < *α*, *p* = 0.0402). The values obtained in R2 are similar to those published by Tao et al. [26] (60% for TN). In turn, Zhou et al. [10] investigated a reactor with GAC having a 0.2 mm particle size and obtained a TN removal efficiency of 72.5%. In the reactor with powdered ceramsite, a very high efficiency of NH_4_^+^–N removal was observed (95.1 ± 12.8%), whereas approximately 78.7 ± 17.0% and 86.6 ± 22.4% of NH_4_^+^–N was eliminated in reactors R1 and R3.

In wastewater treatment systems based on aerobic granular sludge, various mechanisms may be used to remove nitrogen compounds (e.g., assimilation, together with anaerobic ammonium oxidation or simultaneous nitrification and denitrification) [48]. In our research, the removal of nitrogen compounds by simultaneous nitrification/denitrification (SND) was not precluded. The effectiveness of this process in the reactors was evaluated in accordance with the guidelines of Wei et al. [22]. The SND efficiencies of R1, R2, and R3 were 75.7 ± 20.5%, 77.4 ± 7.1%, and 76.8 ± 6.4%, respectively. Note that the values for R1 are unreliable because the overall depletion of ammonium-nitrogen, i.e., the sum of nitrates and nitrites present in the reactor, was not constantly observed. The obtained SND efficiencies are comparable to the literature data. Wei et al. [22] reported that for C/N = 6, the SND efficiency was 94.6% and 92% in a reactor with zeolite and a control reactor, respectively. However, when C/N = 10, the efficiency of this process was lower, at 72.0% and 55.0%, respectively. Adoption of a C/N ratio of 16 in these studies could have affected the TN removal. According to the information provided by literature, it is necessary to develop mature granules for effective denitrification [48]. Moreover, small size of granules may significantly reduce the anoxic zones during the aeration phase [3]. Our own research did not demonstrate that increasing the granulate diameter had any effect on the efficiency of the SND process or the removal of nitrogen compounds.

The influent TP concentration in the three reactors was 9.73 ± 1.05 mg P/L, and the TP loading rate of the GSBRs was 0.035 ± 0.004 g P/(L·d). The TP values in the effluent of reactors R1, R2, and R3 were 4.03–9.36 mg P/L, 2.46–6.76 mg P/L, and 1.98–7.65 mg P/L, respectively (Figure 6e). The lowest TP values were recorded in the outflow from R2. In R3, starting on day 40 of the experiment, the TP concentration in the treated wastewater increased. The average TP removal efficiencies of R1, R2, and R3 were thus 31.5 ± 19.8%, 56.8 ± 9.9%, and 46.5 ± 17.5%, respectively (Figure 6f). Statistical analysis revealed significant differences in TP removal between the reactors (*p* < *α*, *p* = 0.0032).

To date, most papers on support of AGS technologies using powdered materials have ignored the removal of phosphorus compounds. He et al. [4], who used yellow earth to support a reactor operating without an anaerobic phase, obtained TP removal efficiencies of 17–46%. The efficiency of TP removal reported here for reactors with added powdered materials are greater than those presented by He et al. [4]. Our research also did not consider a separate anaerobic phase in the reactor cycle. Tao et al. [26], who used GAC to support a reactor, obtained higher TP removal rates (15–99%). However, they did add a 99-min anaerobic phase to the reactor operating cycle. Analysis of the dynamics of PO_4_^3^^−^–P removal in our research showed a lack of phosphate release, indicating a lack of effective biological dephosphatation, despite the favorable C/P ratio adopted for this process. In view of the above, it can be concluded that the removal of phosphorus compounds could have occurred by precipitation of phosphorus as inorganic phosphate such as Ca_3_(PO_4_)_2_ and MgHPO_4_. Such a mechanism for phosphorus removal in AGS technology has been indicated by, among others, Filali et al. [49] and Manas et al. [50]. There was a high negative correlation between the TP removal and TP loading rate of biomass in the reactors supported by powdered materials.

## 4. Conclusions

The development and maturation of aerobic granules at a low OLR requires a long time period; however, the addition of mineral particles might improve the biogranulation process. This study revealed that powdered ceramsite or limestone affected the formation of granules in reactors under the specified operating parameters (251.9 µm < d in R2 < 783.1 µm and 246.0 µm < d in R3 < 518.9 µm), whereas in the control reactor, complete granulation was not achieved (124.0 µm < d in R1 < 210.0 µm). The use of powdered materials was found to significantly enhance the sedimentation properties of biomass during biogranulation, ensuring a lower rate of biomass elution and a higher biomass concentration in the reactors. Moreover, in R2 and R3 reactors, relationships between the granule diameter and SVI_5_/SVI_30_ or settling velocity were statistically significant (*p* < α, *p* = 0.0105 for R2 and *p* = 0.0221 for R3 or *p* = 0.0000 for R2 and *p* = 0.0000 for R3). The most efficient wastewater treatment (COD: 85.4 ± 5.4%, TN: 56.6 ± 10.2%, and TP: 56.8 ± 9.9%) and strongest effect on the sludge sedimentation properties (resulting in the highest value of MLVSS: 4.12 g/L) were obtained using powdered ceramsite. Moreover, this material acted not only as ballast for the sludge flocs making up the seed sludge, but also as microcarriers of granule-forming biomass. The results showed that, under the specified operating parameters for the control reactor, granule formation could not occur.

An important issue in the assessment of biological wastewater treatment, especially in new technologies, is the accurate recognition of the individual pollution removal processes. Therefore, the next stage of research may be investigation of nitrification, denitrification, and dephosphatation processes, based on profile tests including the determination of COD, NH_4_^+^–N, NO_2_^−^–N, NO_3_^−^–N, and PO_4_^3^^−^–P, in individual phases of the GSBR cycle with the addition of powdered material.

## Figures and Tables

**Table 1:** Physical properties of powdered mineral materials used in previous aerobic granular sludge (AGS) research.

Mineral Material	Specific Surface Area (m^2^/g)	Density (g/cm^3^)	Diameter (µm)	Reference
Basalt	Large *	n.d.	from 212 to 300	[20]
Zeolite	Large *	n.d.	from 150 to 300	[22]
Bivalve shell carrier	Large *	1.45	from 150 to 220	[21,31]
Yellow earth	n.d.	n.d.	above 270	[4]
Ceramsite	5.183	2.6182	to 200	[11,15]

* no numeric value, only term “large” was used. n.d.—no data.

**Table 2:** Physicochemical properties of the applied powdered materials (adopted according to [33]).

Parameter, Units	Powdered Ceramsite (PK)	Powdered Limestone (PL)
Specific surface area (S_BET_), m^2^/g	5.183	1.760
Sphericity/shape	low sphericity	hight sphericity
Granulation: d_90_; d_50_; d_10_, µm	85.279; 24.110; 3.643	189.720; 33.915; 1.865
Apparent density, g/cm^3^	2.6182	2.1949
Chemical composition, mg/g	Ca: 75.90; Fe: 45.15; Mg: 21.61; Si: 216.30	Ca: 691.04; Fe: 1.68; Mg: 4.61; Si: 5.58
Substance leaching, µg/g	Ca: 451.65; Fe: 0.50; Mg: 97.87; Si: 23.01	Ca: 87.75; Fe: 0.21; Mg: 12.67; Si: 7.00
Settling velocity, m/h	approx. 9.0	approx. 12.0

**Table 3:** Technical parameters of the reactors used in this study (adopted according to [15]).

Parameter, Units	R1	R2	R3
Powdered materials	-	ceramsite	limestone
Dosage of powdered material, g/L	-	3.0	3.0
Working height (H) and internal diameter (D), m	0.78 and 0.07 (H/D = 11.1)		
Average daily flow of wastewater, L/d	12.0	12.0	12.0
Amount of wastewater fed during the cycle (ΔV), L	1.5	1.5	1.5
Volumetric exchange ratio (VER), -	0.5	0.5	0.5
Hydraulic retention time (HRT), h	6 ^(^*^)^	6	6
Cycle of operation (3 h)	10 min—feeding without stirring, 160 min—reaction (air supply: 110 L/h), 4 min—settling, 5 min—decantation, 1 min—an idle phase		

^(^*^)^ in order to maintain the MLVSS (mixed liquor volatile suspended solids) in the reactor, it was necessary to control the value of HRT.

**Table 4:** Characteristics of wastewater used in this study.

Parameter, Units	Minimum	Maximum	Average	Standard Deviation
COD, mg O_2_/L	615.0	785.0	717.1	62.6
NH_4_^+^–N, mg N/L	28.8	42.8	39.3	3.4
NO_3_^−^–N, mg N/L	0.51	2.99	1.56	0.61
NO_2_^−^–N, mg N/L	0.002	0.032	0.007	0.008
TN, mg N/L	40.3	50.3	44.0	3.0
PO_4_^3^^−^–P, mg P/L	7.49	9.08	8.27	0.52
TP, mg P/L	8.55	11.6	9.73	1.05
COD/TN ratio	13.8	18.7	16.3	1.6
COD/TP ratio	53.8	89.7	74.9	12.8

**Table 5:** Comparison of biomass parameters in reactors supported by powdered substances with those of a control reactor.

Reactor	OLR g COD/(L∙d)	COD (mg O_2_/L)	SVI (mL/g)	MLSS (g/L)	Diameter of Granules (mm)	References
control	-	min. 1310 max. 5534	-	-	to 0.7 *	[23]
with 1.0 g/L of PAC			38.0	5.0	to 0.6	
control	0.8	200	100.0	approx. 3.0	to 0.25	[24]
with 3.0 g/L of GAC			40.0	approx. 3.4	to 0.6	
control	-	min. 800 max. 2000	47.9	5.45	from 0.6 to 4.0	[22]
with 3.0 g/L of zeolite			34.9	7.36	from 1.0 to 5.0	
control	1.5	503	70.0	3.50	lack of granules	[10]
with 1.0 g/L of GAC			26.0	9.08	0.44	
control	4.0	min. 800 max. 1000	73.0	4.92	approx. 0.2	[25]
with 1.0 g/L of SSM			51.0	8.62	approx. 0.4	
with 20.0 g/L of basalt (SBAR)	3.0–4.0	-	to 32.0	11.23	from 0.3 to 3.7	[20]
with 20.0 g/L of basalt (SBBR)			to 28.0	12.78	from 0.3 to 4.0	
control (35 cm^3^ of anaerobic granules)	2.5–30.0	600	19.0	approx. 15	from 0.3 to 4.0	[21,31]
with 20.0 g/L of BSC			14.0	approx. 10	from 0.5 to 2.0	
with 3.0 g/L of powdered ceramsite	2.1	862.8 ± 31.0	30.1	4.37 **	to 0.96	[11]
	1.0	408.9 ± 12.9	36.9	3.03 **	to 0.29	

PAC—powdered activated carbon; GAC—granular activated carbon; SMM—sewage sludge micropowder; BSC—bivalve shell carrier; SBAR—sequencing batch airlift reactor; SBBR—sequencing batch bubble reactor. * breakup of granules. ** MLVSS (g/L).
